# Beyond direct killing—novel cellular immunotherapeutic strategies to reshape the tumor microenvironment

**DOI:** 10.1007/s00281-022-00962-4

**Published:** 2022-09-27

**Authors:** Duc Huynh, Pia Winter, Florian Märkl, Stefan Endres, Sebastian Kobold

**Affiliations:** 1 Department of Medicine IV, Center of Integrated Protein Science Munich (CIPS-M) and Division of Clinical Pharmacology, Klinikum der Universität München, LMU Lindwurmstrasse 2a, 80337 Munich, Germany; 2 Einheit Für Klinische Pharmakologie (EKLiP), Helmholtz Zentrum München, Research Center for Environmental Health (HMGU), Neuherberg, Germany; 3 German Center for Translational Cancer Research (DKTK), partner site Munich, Heidelberg, Germany

**Keywords:** Cellular immunotherapy, CAR-T cells, Tumor microenvironment, Inflammation, Cytokine, Stroma

## Abstract

The clinical use of cellular immunotherapies is gaining momentum and the number of approved indications is steadily increasing. One class of cellular therapies—chimeric antigen receptor (CAR)-modified T cells—has achieved impressive results in distinct blood cancer indications. These existing cellular therapies treating blood cancers face significant relapse rates, and their application beyond hematology has been underwhelming, especially in solid oncology. Major reasons for resistance source largely in the tumor microenvironment (TME). The TME in fact functionally suppresses, restricts, and excludes adoptive immune cells, which limits the efficacy of cellular immunotherapies from the onset. Many promising efforts are ongoing to adapt cellular immunotherapies to address these obstacles, with the aim of reshaping the tumor microenvironment to ameliorate function and to achieve superior efficacy against both hematological and solid malignancies.

## Introduction

Cellular immunotherapies, encompassing the use of modified autologous or allogeneic immune cells as treatment against disease, have recently advanced to become part of the standard of care in a few types of relapsed and refractory hematological malignancies. Recent evidence suggests that they might even progress to earlier lines of treatment at least in some indications, highlighting the untapped potential of these approaches.

Overall, cellular immunotherapies have so far employed dendritic cell (DC), T cells, or natural killer cells (NK) into patients for treatment of disease. DC vaccines, which utilize mature dendritic cells or monocyte-derived dendritic cells derived from patient blood, harness the natural role of the dendritic cell in antigen presentation and T cell licensing to target cancer. Dendritic cells are pulsed with tumor-associated antigens and neoantigens that would then be presented to T cells in lymph nodes to induce cytotoxic lymphocyte priming and polarization to mount a specific immune response. Various DC vaccines have shown promising safety and efficacy in clinical trials against pediatric solid tumors and other forms of solid tumors [1–3]. These efforts in developing DC vaccine approaches culminated in FDA approval of Sipuleucel-T, a DC vaccine for the treatment of prostate cancer [4]. While Sipuleucel T did improve survival in prostate cancer patients, adoption of this therapy in clinics has been limited over questions of clinical efficacy and cost [5]. In fact, marketing authorization was even withdrawn in the European Union. While NK cell therapy to date has remained investigational only, most efforts and advances in cellular therapy have revolved around T cell-based therapeutics. These therapies can be subclassified into tumor infiltrating lymphocytes (TIL), chimeric antigen receptor (CAR)-T cells, and recombinant T cell receptor (TCR)-T cells. TILs are heterogeneous populations of immune cells, mostly composed of T cells, that are extracted from a patient’s own cancer as they are presumed to have high specificity against those tumors [6]. After expansion, the cells are reintroduced into the patient in a therapeutic intention. Such treatment was initially sought for in melanoma treatment, where high remission rates were observed [7]. The burden of generation and high variability of the product made up for an uneven comparison to checkpoint blockade and dismissed TIL therapy for a while. Recently, autologous TIL treatment after immune checkpoint blockade (ICB) failure demonstrated robust remission rates in a significant share of melanoma patients, bringing the concept back to clinical investigations [7].

Meanwhile, TCRs and CARs are receptors genetically engineered into T cells isolated from patient blood and reintroduced into the patient to target cancers. TCRs used are derived from natural sequences that bind the desired antigen and behave similarly to normal TCRs [8]. In contrast, CARs are fully synthetic receptors not naturally found and consist of an antibody-derived single chain variable fragment (scFv) combined with intracellular T cell receptor signaling domains only (so called 1st generation CAR) or with one or more intracellular domains of costimulatory molecules to further activate the T cell when antigen is bound (so called 2nd and 3rd generation CAR) [9]. The major difference is HLA restriction in the case of TCRs and lack thereof for CARs. Both strategies activate the T cell and induce cytotoxicity and proliferation when in contact with tumor cells [6, 9]. While TCRs have demonstrated only anecdotal evidence and a slow developmental pace [10], CAR usage has culminated into six FDA approvals for T cell-based therapies—idecabtagene vicleucel and ciltacabtagene autoleucel targeting B cell maturation antigen (BCMA) in multiple myeloma, and lisocabtagene maraleucel, tisagenlecleucel, brexucabtagene autoleucel, and axicabtagene ciloleucel targeting CD19 in a variety of B-cell lymphomas and leukemias [11]. There are also efforts also to adapt CAR engineering to NK cells, which would confer an antigen-specific tumor killing capacity to cells optimized for serial killing in efforts to induce efficient anti-tumor activity [12], and to γδ T cells, which have shown antigen cross-presentation capability and favorable persistence phenotypes [13, 14]. In hematological malignancies, CAR-T cells have achieved unparalleled clinical results as treatment for relapsed and refractory patients with otherwise poor prognosis [15]. Despite successes and high response rates, patients undergoing CAR-T cell therapy often experience relapse after only weeks or months, demonstrating that current forms of therapy must be improved [16]. At the same time, CAR-T cell therapies in their current form have shown modest or negligible efficacy in clinical trials for a range of solid tumors [17, 18]. Overall, ongoing issues of relapse in hematological malignancies and inefficacy in treating solid tumors can be routed back to a few main obstacles: antigen loss, low levels of immune cell infiltration, and the effects of an intensely immunosuppressive tumor microenvironment (TME) [17, 18]. As several of these aspects have been extensively reviewed by us and others in the past [19], this review will specifically focus on the obstacles presented by the TME. We will summarize current efforts in cellular immunotherapies to affect and change the TME to be more therapy permissive, with the aim of enabling increased therapeutic efficacy across indications.

## The immunosuppressive tumor microenvironment

**Fig. 1:**
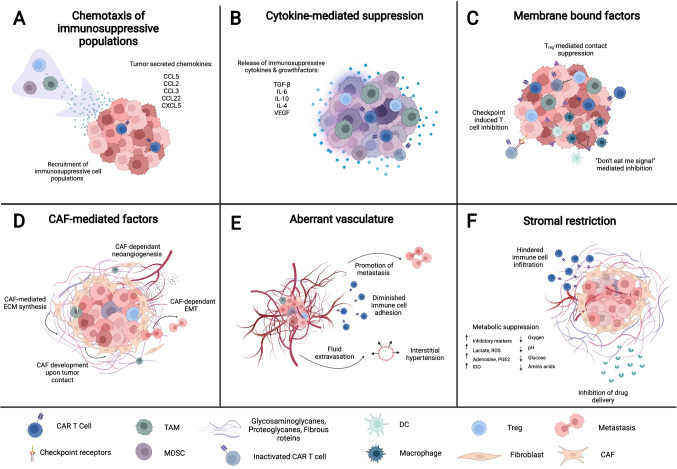
Mechanisms of TME suppression of anti-tumor cellular therapies. (**A**) Recruitment of immunosuppressive TAMs, MDSCs, and T-regs by chemokine gradients secreted by tumor cells. (**B**) Immunosuppressive cytokine milieu secreted by tumor cells, immunosuppressive cells, and CAFs exhaust and inactivate infiltrated adoptive cells. (**C**) Membrane-bound mechanisms of TME suppression mediated by tumors and immunosuppressive cells. (**D**) Pro-tumorigenic and metastatic functions mediated by CAFs. (**E**) Inhibition of cellular therapies by the development of aberrant vasculature. (**F**) Stromal exclusion of immune infiltration and suppression of infiltrated cells

The tumor microenvironment—comprises malignant cells together with their immediate environment consisting of immune cells, fibroblasts, extracellular matrix (ECM), blood vessels, and secreted factors. The TME can support the survival and proliferation of tumor cells and tumor-associated cell populations and thus contributes a major share to cancer hallmarks (Fig. 1). Crucially, the TME suppresses the ability of cellular therapies to normally function through induction of inhibitory signals as well as forming physical barriers preventing contact with tumor cells in both solid tumors and hematological malignancies [20]. These immunosuppressive aspects relevant to cellular therapies are the focus of this section. Suppressive mechanisms embedded in the TME can be subdivided into chemokine-induced migration of immunosuppressive cells, cytokine-mediated suppression, membrane-bound and contact suppression, cancer-associated fibroblast-mediated factors, stromal restriction, and aberrant vasculature (Fig. 1).

Chemokines released by tumor cells recruit and retain other populations of immunosuppressive cells into the tumor microenvironment (Fig. 1A). T-regulatory cells (T-reg) are recruited by tumor-secreted chemokines C–C motif ligand (CCL)5, CCL17, and CCL22 [21]. Chemokines such as CCL2, CCL5, and C-X-C motif chemokine (CXCL)5 recruit myeloid-derived suppressor cells (MDSC) [22], a heterogenous though poorly defined population of myeloid cells that are most clearly related by their myeloid lineage and suppressive capacity [22]. Tumor-associated macrophages (TAM), a suppressive type of macrophage closely related to M2 macrophages, derive from tumor-associated monocytes that migrate to tumors via CCL2 and CCL3 gradients [23]. Other varieties of recruited cells such as mast cells and neutrophils also contribute to suppression [24, 25] while cells such as bone-marrow-derived mesenchymal stem cells (MSC) additionally contribute to the development of the TME [26]. Tumor cells and recruited immunosuppressive cells release cytokines such as interleukin (IL)-10, transforming growth factor–beta (TGF-β), IL-4, and the enzyme indoleamine 2,3-dioxygenase 1 (IDO-1) that contribute a cytokine milieu that further suppresses infiltrated immune cells [20] (Fig. 1B). In addition to these soluble factors, tumors, MDSCs, TAMs, and T-regs directly suppress immune function by upregulating inhibitory signaling molecules on their membrane, including the well-known checkpoint molecules programmed cell death protein 1 (PD-1) and cytotoxic T-lymphocyte-associated protein 4 (CTLA-4), among others [20, 27] (Fig. 1C). Tumor cells furthermore prominently express “don’t eat me” signals such as CD47 and CD24 to escape immunosurveillance by the innate immune system[28–30], and intratumoral T-regs can mediate antigen-dependent and -independent inhibition of immune cells [21]. Another intratumoral cell type, cancer-associated fibroblasts (CAF), are a highly heterogeneous population of activated fibroblasts that form around tumors and further shape the tumor microenvironment [31] (Fig. 1D). Though the mechanisms of CAF development are not fully understood, it is clear that these cells both shape and are shaped by the environment around tumors [32, 33]. Several intra-tumoral factors such as cell–cell contact with tumor cells, DNA damage, TGF-β secretion, and physiological stress have been shown to induce the transformation of normal tumor-adjacent fibroblasts and tumor-infiltrated MSCs into CAFs [26, 33]. CAFs then support tumor growth through the promotion of angiogenesis via the secretion of a variety of growth factors such as VEGF, PDGF, EGF, FGF2, FGF5, GDF15 and the secretion of immunosuppressive cytokines including TGF-β, IL-6, CXCL12, CCL2, LIF, and GAS6 [33, 34]. CAFs furthermore are crucial in the degradation and formation of the extracellular matrix (ECM) of the TME, a network of proteoglycans and glycoproteins that provides structural and mechanical support to cells and tissues. This CAF remodeling function has also been shown to be important for tumors undergoing endothelial-mesenchymal transitions (EMT) crucial for metastasis [35]. Altogether, CAFs and infiltrated immunosuppressive cells in turn constitute a protective layering of cells and ECM around the tumor known as the tumor stroma [36]. The stroma physically functions as a barrier excluding immune cells from accessing the tumor, while further metabolically suppressing immune cells by depleting vital acellular components such as amino acids, glucose, and oxygen from the TME [37, 38] (Fig. 1F). The stroma has also been shown to inhibit the delivery of anti-cancer drugs and therefore contributes to resistance against certain therapies [35, 39, 40]. Finally, aberrant vasculature needed by malignant cells to proliferate and metastasize poses an additional barrier confronting CAR-T cells upon entrance into the TME (Fig. 1E). This irregular vasculature is promoted by a combination of angiogenic signaling from tumor cells themselves and by tumor-associated cells mentioned previously. The vessels are not only characterized by their disorganized manner, but also through an upregulation of endothelial molecules and a highly permeable endothelial membrane, consequently limiting the trafficking of immune cells within the malignant tissue via interstitial hypertension and interference with T cell adhesion [41]*.* Along these lines, the TME profoundly regulates and suppresses immune responses and appears as the aspect to beat to enable cell therapy efficacy.

## Cellular therapies to remodel the TME

**Fig. 2:**
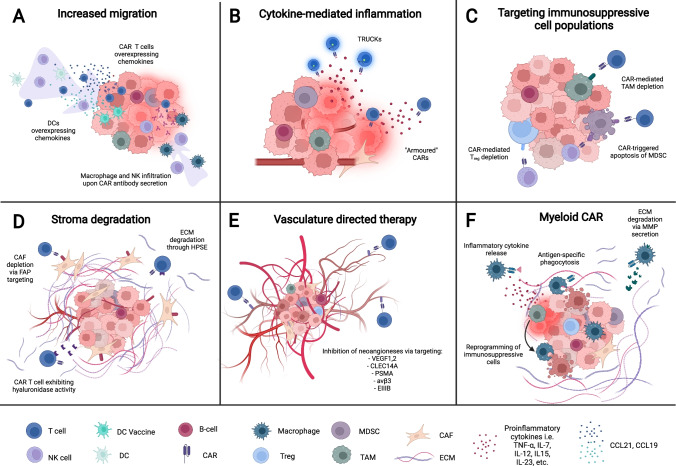
Strategies incorporated into cellular therapies to remodel the TME. (**A**) Increased migration of endogenous and adoptive cells via cellular therapies engineered to overexpress inflammatory chemokines and secreted antibodies. (**B**) Overexpression of inflammatory cytokines reprograms immunosuppressive populations, converts immune-restricted tumor to an immune-permissive tumor. (**C**) CAR-mediated depletion of MDSCs, TAMs, and T-regs via direct targeting. (**D**) Degradation of stroma components via enzymes engineered into cellular therapies or direct targeting by CARs. (**E**) CAR targeting of angiogenic markers inhibits tumor neoangiogenesis. (**F**) CAR-M- and CAR-P-mediated tumor phagocytosis, and the inflammatory and physical remodeling of the TME

Many insights have already been gained into improving existing cellular therapy. For instance, application via intratumoral injection leads to increased efficacy of adoptive CAR-T cells in certain tumor indications by physically circumventing the issue of TME exclusion and adoptive cell infiltration [42, 43]. To further address the issues of cellular therapies in tumors, recent therapeutic concepts have incorporated elements designed to specifically counteract various aspects of the TME (Fig. 2), with some reaching the clinical trial stage (Table 1). Because the range of these approaches varies widely, the scope of the present review will focus on concepts that are specifically incorporated into the cellular therapy. Combinatorial approaches with application of secondary agents have already been extensively reviewed [44]. We emphasize novel approaches that have been shown to affect and change obstacles presented by the TME, covering topics such as increasing intratumoral immune cell infiltration, changing the cytokine environment within the tumor, elimination of suppressive immune cell populations, removal of physical barriers presented by the stroma, and targeting of the tumor vasculature (Fig. 2). Additionally, we focus on concepts that remodel the TME specifically rather than those enhancing cellular therapy function only. Such approaches have been shown to have higher potential of engaging concerted endogenous immune responses against the tumor and have been shown to pair well with standard of care ICB [45–48].

**Table 1:** Clinical trials

Drug type	Mechanism	Target	Start	Phase	Status	Trial number
Increasing intratumoral immune infiltration						
DC vaccine	Adenovirally transduced CCL21 DC vaccine	Melanoma	2008	I	Completed	NCT00798629
DC vaccine	Adenovirally transduced CCL21 DC vaccine	NSCLC	2011	I	Completed	NCT01574222
DC vaccine	Adenovirally transduced CCL21 DC vaccine and pembrolizumab	NSCLC	2019	I	Recruiting	NCT03546361
CAR T cells	Anti-GPC3 or TGF- β CAR T cells overexpressing CCL19-IL7	HCC	2017	I	Recruiting	NCT03198546
CAR T cells	CAR T cells overexpressing CCL19-IL7 (single and dual targeting of: Integrin β7, BCMA, CS1, CD38 and CD138)	RRMM	2018	I	Recruiting	NCT03778346
CAR T cells	Anti-Nectin4 + CAR T cells overexpressing -IL7 and IL-12 or CCL19	Nectin-4 + malignant solid tumors	2019	I	Recruiting	NCT03932565
CAR T cells	Anti-CD19 CAR T cells overexpressing CCL19-IL7	B-cell lymphoma	2019	II	Unknown	NCT03929107
CAR T cells	Anti-CD19 CAR T cells overexpressing CCL19-IL7 and PD1 monoclonal antibody	DLBCL	2020	I	Recruiting	NCT04381741
CAR T cells	Anti-CTLA-4/PD-1 expressing EGFR-CAR-T	EGFR + advanced solid tumors	2017	I,II	Unknown	NCT03182816
Cytokine-mediated inflammation						
CAR T cells	IL-12 armored anti-Nectin4 CAR T cells	Nectin-4 + malignant solid tumors	2019	I	Recruiting	NCT03932565
CAR T cells	IL-12 armored anti-MUC16 CAR T cells	MUC16 + solid tumors	2015	I	Active, not recruiting	NCT02498912
CAR T cells	IL-12 armored anti-EGFR CAR T cells	Metastatic CRC	2018	I	Unknown	NCT03542799
CAR T cells	IL15 armored anti-GPC3 CAR T cells	GPC3 + solid tumors	2021	I	Recruiting	NCT04377932
CAR T cells	IL15, IL21 armored anti-GPC3 CAR T cells	GPC3 + solid tumors	2023	I	Not yet recruiting	NCT04715191
CAR T cells	IL15 armored anti-GPC3 CAR T cells	HCC	2021	I	Recruiting	NCT05103631
CAR T cells	IL15 armored anti-GD2 CAR T cells	Neuroblastoma, osteosarcoma	2019	I	Recruiting	NCT03721068
CAR T cells	IL18 armored anti-CD19 CAR T cells	NHL, CLL	2021	I	Recruiting	NCT04684563
Targeting immunosuppressive axes						
CAR T cells	Anti- CD123 CAR T cells	Refractory AML	2015	I	Terminated	NCT02623582
CAR T cells	Anti- CD123 CAR T cells	AML	2019	I	Withdrawn	NCT04106076
CAR T cells	Anti- CD123 CAR T cells	BPDCN	2017	I	Terminated	NCT03203369
CAR T cells	Anti- CD123 CAR T cells	AL, AML	2019	I	Terminated	NCT03672851
CAR T cells	Anti- CD123 CAR T cells	Refractory AML	2018		Terminated	NCT03473457
CAR NK cells	Anti-NKG2D CAR NK cells	Solid tumors	2018	I	Unknown	NCT03415100
CAR NK cells	Anti-NKG2D CAR NK cells	Refractory metastatic CRC	2022	I	Recruiting	NCT05213195
CAR NK cells	Anti-NKG2D CAR NK cells	Relapsed or refractory AML	2022	I	Recruiting	NCT05247957
Physical barriers						
CAR T cells	Anti-FAP CAR T cells	MPM	2015	I	Completed	NCT01722149
CAR T cells	FAP/Nectin-4 CAR T cell	Nectin-4 + malignant solid tumors	2019	I	Recruiting	NCT03932565
CAR T cells	Anti-VEGFR-2 CAR T cell + cyclophosphamide, aldesleukin, fludarabine	Metastatic cancer, metastatic melanoma, renal cancer	2010	I,II	Terminated	NCT01218867
Myeloid CARs						
CAR macrophage	Anti-HER2 + CAR macrophages	Breast cancer	2021	I	Not yet recruiting	NCT05007379
CAR macrophage	Anti-HER2 + CAR macrophages	HER2 + solid tumors	2021	I	Recruiting	NCT04660929

## Increasing intratumoral immune infiltration

Chemokines are mediators signaling for directed migration towards the source they originate from (a concept also known as chemotaxis). Chemokines orchestrate the localization of cells within the body and accordingly play a crucial role in the trafficking of immunosuppressive cells to the TME [49, 50]. However, they are also involved in intratumoral migration of proinflammatory and anti-tumoral immune cells such as CD1c + dendritic cells, T cells, and NK cells. In such situations, infiltration of these populations within the TME has been strongly correlated with better cancer patient prognosis [51–53]. We previously reviewed the role and therapeutic utilization of chemokines in cancer immunotherapy [50]. Based on such observations, infiltration of therapeutic immune cells can either be increased by engineering therapeutic cells for desired chemokine expression or by introducing chemokine receptors matching intratumoral chemokine gradients. The later does not impact the TME directly and thus is not part of this review, but a current summary can be found here [50].

Chemokine engineering into therapeutic immune cells has been incorporated into cellular therapies with the goal of increasing the migration of both endogenous inflammatory cells and also adoptively transferred cells into the tumor (Fig. 2A). The chemokine CCL21 was adenovirally engineered to be secreted from an anti-tumor DC vaccine to elicit signaling through CCR7 expressed on endogenous dendritic and T cells. As the DC vaccine is injected intratumorally, this establishes a gradient of CCL21 that promotes infiltration of those endogenous cells into the tumor, which has been shown to increase synergistic anti-tumor effects between adoptive and endogenous cells [54]. This approach has led to clinical trials in melanoma (NCT00798629), and in non-small cell lung cancer (NCT01574222) both as monotherapy and in combination with pembrolizumab (NCT03546361). The first results from these studies demonstrated increased immune infiltration and hints towards a modest increase in survival [50, 55]. Along the same lines, CAR-T cells have also been adapted to exploit chemokine signaling axes. While CAR-T cells are injected intravenously, the extracellular domain of the CAR construct confers an antigen-specific binding capacity that anchors the CAR-T cell within antigen-positive tumors [9]. Preclinically, CAR-T cells against CLDN18.2 were enhanced to overexpress CCL21 and IL-7 [56], with CCL21 here serving the same purpose of enhancing intratumoral migration of DC and T cells [50, 56]. In another approach, CAR-T cells have been enhanced by dual overexpression of CCL19 and IL-7, with CCL19 here being another ligand of CCR7 which leads to increased migration of endogenous immune cells into the tumor. CCL19-IL7 overexpression has been utilized in multiple CAR-T cells in numerous clinical trials: a phase I trial utilizing anti-GPC3 CARs against hepatocellular carcinoma (NCT03198546), a phase I trial utilizing multiple CARs against relapsed-refractory multiple myeloma (NCT03778346), a phase I trial utilizing anti-Nectin-4 CAR against Nectin-4-positive advanced-stage solid tumors (NCT03932565), a phase II trial utilizing CD19 CARs against B cell lymphomas (NCT03929107), and a phase I trial utilizing CD19 CARs in combination with tislelizumab against B cell lymphomas (NCT04381741) [50, 57, 58]. For the trials having reported data, safety of the approach was demonstrated along with early signs of activity [50].

Another related strategy to increase immune infiltration is endogenous activation and proliferation, ultimately leading to higher anti-tumor effector cell numbers at the tumor site. In this sense, T cells can be genetically engineered to express immune checkpoint inhibitors, with anti-PD-1 or anti-PD-L1 antibodies being the most utilized avenue [59–61]. Anti-PD-1 antibodies typically lead to an increased activation and expansion of CAR and endogenous T cells, thereby mediate an enhanced cytolytic activity [59]. Along these lines, CAR-T cells secreting anti-PD-L1 Ig1 isotype antibodies capable of mediating ADCC simultaneously increased the amount of tumor-infiltrating NK cells [61]. Similarly, CAR-T cells secreting nanobodies targeting the prominent “don’t eat me” signal CD47 induced enhanced infiltration and activation of macrophages in the tumor with limited systemic toxicity [62]. So far, only anti-CTLA-4- and anti-PD-1-secreting EGFR-specific CARs were tested in a phases I and II clinical trial in patients with EGFR + advanced solid tumors (NCT03182816). In total, strategies to equip cellular therapies to additionally increase migration of beneficial immune cells into the TME significantly increase their efficacy. While promising, other suppressive mechanisms of the TME remains relatively functional, necessitating further strategies to address them to further enable effective therapy.

## Cytokine-mediated inflammation of the TME

The TME is typically rich in cytokines such as IL-1, favoring expansion and polarization of immune suppressive cell populations of myeloid and lymphoid origin [63]. In contrast, proinflammatory or T cell supporting cytokines are either absent or scavenged by immune suppressive populations. Several previously mentioned cellular therapy concepts that mediate migration through chemokine modulation also synergistically employ cytokine co-expression, such as the CCL19-IL7 and CCL21-IL7 CAR where IL-7 promotes proliferation and survival of T cells [57, 58]. These represent only a fraction of the cellular therapies that aim to modulate levels of cytokines in the tumor environment, thereby either enhancing the function of the cellular therapies themselves or creating a more permissive TME for their efficacy (Fig. 2B). Many concepts aim to directly increase levels of cytokines that enhance T cell function. T cells redirected for antigen-unrestricted cytokine-initiated killing (TRUCK) are engineered to express cytokines under the control of an NFAT or similar promoter which are activated upon CAR/TCR engagement [64]. “Armored” CARs in contrast constitutively express these cytokines for permanent enhanced cellular function [65, 66]. Though not exhaustively listed due to the magnitude of research, examples of cytokines targeted for co-expression on these cellular therapies include IL-7 [67], IL-12 [66, 68–70], IL-15 [71–73], IL-18 [74–76], IL-21 [77, 78], IL-23 [79], IL-24 [80], IL-33 [81], and IL-36 g [82]. These cytokines have a range of effects that include signaling T-cell survival, improving T-cell proliferation, and promoting their differentiation into further subtypes. CAR-T cells overexpressing these cytokines thus demonstrate a range of augmented functions including enhanced tumor killing, enhanced secretion of secondary cytokines, improved survival and proliferation, and resistance to immune suppression. In addition, effects of the cytokine modulation on the TME include downregulation of T-regs, reprogramming of TAMs and MDSCs from immune suppression to tumor engagement, and activation of endogenous immune cells [68, 76, 83]. In terms of development, several cytokine-overexpressing constructs have progressed to clinical trials. IL-12 engineering in anti-Nectin-4 CAR-T cells has progressed to a phase I clinical trial against Nectin-4 + solid tumors (NCT03932565), as well as with anti-MUC16ecto CAR-T cells against ovarian cancers (NCT02498912), and with anti-EGFR CAR-T cells against metastatic colorectal cancer (NCT03542799), all ongoing. Furthermore, IL-15 and IL-21 armored anti-GPC3 CAR-T cells have progressed to several ongoing phase I clinical trials against GPC3 + pediatric solid tumors (NCT04377932, NCT04715191), hepatocellular carcinoma (NCT05103631), neuroblastoma (NCT03721068), and osteosarcoma (NCT03721068). Moreover, IL-18 overexpressing anti-CD19 CAR-T cells are currently utilized in a phase I trial against non-Hodgkin lymphoma and chronic lymphocytic leukemia (NCT04684563). Overall, cellular therapy concepts engineered to induce cytokine-mediated inflammation via overexpression can affect broad ranging TME shifts and demonstrate encouraging improvements over their current clinical form. However, cytokines alone may not suffice to overcome immune suppression in the TME, which might require dedicated targeting.

## Targeting immunosuppressive axes in the TME

The aforementioned strategies remodeling the cytokine environment in the TME indirectly affect TAMs, MDSCs, and T-regs by antagonizing their suppressiveness or reversing their suppression program. However, other cellular therapy approaches aim to directly target these immunosuppressive populations for elimination or remodeling (Fig. 2C). For example, CAR-T cells against CD123, target both Hodgkin lymphoma (HL) cells as well as TAM that prominently present in the tumor microenvironment of that indication, as similar expression is found on both cell populations [84]. These CAR-T cells recognize and kill both HL cells and TAMs, which leads to resistance against suppression, and sustained clearance in in vivo models [84]. Targeting of CD123 thus advanced to phase I clinical trials. Interestingly, many trials seem to have been stopped for non-clinical reasons with unclear results (NCT02623582, NCT04106076, NCT03203369), while one did not achieve expected therapeutic effects (NCT03473457). Another trial was terminated for adverse effects (NCT03672851). Further approaches feature T cells and NK-92 cells (an immortalized natural killer cell line that can be employed for therapy) engineered with a CAR against colony-stimulating factor 1 receptor (CSF1R), a receptor expressed on both TAMs in the tumor microenvironment and M2 macrophages. Albeit promising, the study remains in the early proof-of-concept phase [85]. A similar approach utilizing CAR-T cells targets folate receptor β (FRβ) on TAMs of ovarian cancer. These CAR-T cells eliminate TAMs in the TME and led to an increase in endogenous immune cells and prolonged survival of mice [86]. Of particular interest, these cells enabled the enhancement of a secondary tumor targeting anti-MSLN CAR-T cells in further studies. Clinical translation of the concept is pending as well. MDSCs, another immunosuppressive population in the TME, can be also targeted by cellular immunotherapy. Tumor necrosis factor-related apoptosis induced ligand-receptor 2 (TR2) expression on MDSCs can trigger apoptosis when bound to its ligand TRAIL. To exploit this axis, a chimeric costimulatory receptor consisting extracellularly of the scFv of a TR2 agonist antibody and intracellular 4-1BB was co-expressed on anti-Muc1 CAR-T cells. There, it converts normal suppressive CAR-T cell interactions with MDSCs into apoptotic signals for the MDSCs and a costimulatory signal for CAR-T cells [87]. When utilized against an in vivo breast tumor model with additional exogenous MDSCs, the construct enhanced Muc1 CAR anti-tumor activity despite the presence of immunosuppressive MDSCs [87]. Similarly, a novel NKG2D-CD3z chimeric activating receptor was engineered into NK cells, which then targeted NKG2D on MDSCs [88]. In MDSC-negative models, the construct delayed tumor growth only modestly. However, the NKG2D-based construct induced significant anti-tumor activity and displayed promising synergy with CAR-T cells in models with exogenous MDSCs [88]. While most of the aforementioned MDSC-targeting concepts have not yet advanced to the clinical trial stage, NKG2D-targeting NK cells have advanced to clinical trials targeting metastatic solid tumors (NCT03415100), metastatic, refractory colorectal cancer (NCT05213195), and relapsed, refractory AML (NCT05247957), all currently in the recruitment phase. It should be noted however, that these mentioned NKG2D-targeting NK trials were initiated for NKG2D expression on target tumor cells, though effective function on intratumoral MDSCs may very well influence trial results. Finally, T-regs can also be targeted by cellular immunotherapies in efforts to reduce the immunosuppressiveness of the TME. An anti-CD25 CAR was employed in NK-92 cells to target intratumoral T-regs in preclinical studies [89]. As CD25 is highly expressed on endogenous T-regs and activated T cells, this concept instead incorporated NK cells to reduce predicted on-target, off-tumor CAR-T cell-induced toxicity [89]. Though the anti-tumor function of these CAR-NK cells was promising, no conclusive result on increased safety and reduced toxicity was determined. Ultimately, targeting intratumoral suppressive cells has shown to be an efficacious approach, though special care needs to be taken to achieve effective targeting without inducing harmful off-tumor adverse effects.

## Removing physical barriers to cellular immunotherapy

Significant obstacles of cellular immunotherapy in solid tumors do not only arise from immunosuppressive cell populations and a lack of immune cell activation within the TME but are additionally characterized by the establishment of CAF-mediated tumor stroma as a physical barrier, diminishing cellular therapy effectiveness. Along these lines, therapy can be enhanced if either the cellular components creating stroma are depleted or acellular stromal components are digested (Fig. 2D). Fibroblast activation protein (FAP), a transmembrane serine protease/type 2 dipeptidyl peptidase, has been shown to be one of the preponderantly expressed surface markers of most CAF populations and correlates with poor clinical outcome in various carcinomas [90, 91]. With this rationale, anti-FAP CAR-T cells were developed to target CAFs, with multiple concepts aiming to treat malignant pleural mesothelioma (MPM) in human and murine models [92, 93]. These efforts advanced into a now-completed phase I clinical trial against MPM, demonstrating good safety data and encouraging efficacy (NCT01722149) [94]. Similarly, pre-clinical studies on dual therapy with anti-FAP CAR targeting CAFs and anti-Ephedrin A2 (EphA2) targeting human lung cancer demonstrated synergy otherwise not achieved with either therapy alone [93]. Dual therapy is also being attempted in an ongoing clinical trial with CARs targeting both FAP and Nectin-4 in Nectin-4-positive malignant solid tumors (NCT03932565). It should be noted that FAP targeting does show discordant results in terms of toxicity with reports of severe cachexia and bone marrow hypoplasia in mice upon FAP-directed CAR-T cell administration, thus urging caution in clinical trials [95].

A different strategy to surmount the desmoplastic stroma of the TME and enable superior immune cell infiltration is the direct targeting of fibrous proteins, glycosaminoglycans, and proteoglycans composing the ECM via matrix-degrading components. Heparanase (HPSE) is an enzyme that decomposes heparan sulfate proteoglycans and has been found to be insufficiently expressed in CAR-T cells, thereby limiting their capacity to infiltrate stroma-rich tumors [96]. Equipping anti-GD2 CAR-T cells with HPSE enabled superior ability to degrade the ECM, resulting in increased T cell infiltration and antitumor efficacy in human neuroblastoma and melanoma models [96]. Another concept engineered anti-mesothelin CAR-T cells to secrete the hyaluronidase PH20. PH20-expressing CAR-T cells degraded hyaluronic acid within the ECM and resulted in enhanced transmigratory efficacy in vitro and diminished tumor growth in two different xenograft gastric cancer models in mice [97]. Despite possible hints at toxicity with specifically targeting FAP, targeting the CAF-mediated stroma as a whole remains a viable approach being tested preclinically and clinically both as standalone therapy and in combination with other tumor-targeting therapies.

## Targeting aberrant tumor vasculature

Various strategies have also been developed to address the secondary issue of physical tumor-exclusion—aberrant and dysfunctional vasculature that leads to poor T cell infiltration (Fig. 2E). One strategy conceived to address this are CAR-T cells engineered to target VEGFR-1, a receptor expressed on both tumor cells and tumor vasculature. These cells achieved both slowed tumor progression and inhibition of neo-angiogenesis in human models of metastatic lung cancer [98]. Another concept transducing T cells to co-express both an anti-VEGFR-2 CAR and inducible IL-12 augmented the tumor clearance in multiple in vivo models by targeting tumor vasculature as well as decreasing VEGFR-2-expressing MDSC subsets [99]. Despite promising preclinical data, the first clinical trials with anti-VEGFR-2 CAR-T cells in various metastatic cancers have yet to show robust efficacy, with only one patient out of 24 experiencing a partial response (NCT01218867). Additional targets, such as TEM8 have been utilized to further evaluate vasculature-directed CAR-T cell therapy in patient-derived xenograft and pulmonary metastatic triple-negative breast cancer (TNBC) cell line-derived xenograft models, resulting not only in ECM clearance and inhibition of neovascularization, but also suppression of associated breast cancer stem cells [100]. The selection of CLEC14A in models of pancreatic and lung carcinomas, as well as the human prostate-specific membrane antigen (PSMA), expressed on malignant vasculature of various tumors or the neoangiogenesis-associated αvβ3 integrin, demonstrated further promising antigen options for future CAR T-cell constructs [101–103]. Lastly, the use of nanobody-based CAR-T cell therapy with the variable domain of the heavy chain-only antibody directed against EIIIB, a fibronectin splice variant found in tumors and during angiogenesis, has proven to inhibit tumor growth in melanoma- and colon carcinoma-bearing mice [104]. These studies point to the promising future of targeting tumor vasculature, though clinical efficacy of these approaches has yet to be fully demonstrated.

## Myeloid CAR for broad tumor and TME targeting

Recent efforts to remodel the tumor microenvironment with cellular therapies have advanced with the advent of engineering CARs or CAR-like receptors on myeloid lineage cells, notably on macrophages and in some instances dendritic cells (Fig. 2F). Traditionally associated with the innate immune system, these macrophages and dendritic cells are known to be prominent antigen presenting cells (APC) at the forefront of infection and cancer surveillance [105–107]. During the course of infection and other aberrant stimuli, these cells sense pathogen-derived proteins and molecules, and their resulting activation leads to the mobilization of an immune response, the release of inflammatory cytokines including type I interferons (IFN), and presentation of foreign antigen [108]. In the cancer setting, the TME of many cancer types features this type I IFN signature from APCs [109]. Typically, however, tumors adversely suppress this immune response via expression of “don’t eat me” signals, immunosuppressive cytokines, and in the case of TAMs, reprogramming of this population of cells towards pro-tumorigenic function, leading to escape from tumor immune surveillance [28, 110]. To hijack this tumor suppression function, chimeric antigen receptors for phagocytosis (CAR-P) have been proposed. These CAR-like receptors encompass antigen-specific scFv extracellularly and either the multiple EGF-like-domains 10 (Megf10) intracellular domain or the common ɣ subunit of Fc receptors (FcRɣ). CAR-Ps can be engineered onto human monocyte-derived macrophages (MDM) for antigen-specific phagocytosis of target cells and inflammation [111]. Another concept engineering MDMs with a first-generation CAR targeting HER-2 has similar potent phagocytotic anti-tumor activity against breast cancer. These CAR-macrophages (CAR-M) maintain an inflammatory M1 phenotype intratumorally, and further demonstrate therapy-induced remodeling of the TME with increased expression of pro-inflammatory markers on intratumoral immune populations in humanized murine models [112]. CAR-Ms have proceeded to two ongoing clinical trials in patients suffering from HER2 + breast cancer (NCT05007379) and HER2 + solid tumors (NCT04660929). Both CAR-Ps and CAR-Ms demonstrated the capacity to cross-present antigen on MHC-I, adding synergistic potential for these cells to further mobilize an endogenous CD8 T cell-mediated immune response against tumors [111, 112]. CAR engineering in macrophages can be further augmented by addition of matrix metalloproteinases (MMP) for increased ECM degradation. Though macrophages are endogenously a significant source of MMPs, these engineered macrophages demonstrated enhanced inhibition of tumor growth and led to elevated T cell infiltration in human breast cancer models [113]. While myeloid CAR has demonstrated promising results, questions remain over intratumoral persistence as well as the stability of the M1 phenotype, the loss of which may instead cause the therapy to promote tumor growth [114].

## Conclusions and future perspectives

While cellular therapies have come a long way to become an established method of cancer treatment, their future potential for increased efficacy in hematological malignancies and even meaningful efficacy in solid tumor indications largely relies on their ability to address the obstacles posed by the TME. This review highlights the current considerable efforts that have been made to improve various aspects of cellular therapies to reshape the TME to become more permissible for treatment: novel concepts that target immunosuppressive cell populations, inflame an immune-restricted tumor, and address chemotactic signaling and physical barriers excluding immune infiltration. The improvements made on these therapies have been shown to lead to superior anti-tumor efficacy compared to more standard cellular therapies. Moreover, many cellular therapies covered here that target the TME as an entity have been combined with other treatments that primarily target the tumor, forming a dual-pronged targeting approach that in many cases shows synergistic efficacy. Additionally, concepts incorporating combinatorial improvements that address multiple TME obstructions show further enhanced function, demonstrating additionally the promising and synergistic nature of reshaping the tumor microenvironment, and hints at robust effects for combinatorial treatments with existing therapies such as ICB. Promising pre-clinical and clinical results of many of the therapies listed bode well for the application of cellular immunotherapies for the treatment of both hematological and solid tumors in the future.
